# Association and Discriminative Performance of a Body Shape Index (ABSI) for Cardiometabolic Disease in a Korean Population

**DOI:** 10.3390/healthcare14162463

**Published:** 2026-08-10

**Authors:** Myong-Won Seo, Joon Young Kim

**Affiliations:** 1 Department of Sports and Leisure Studies, College of Physical Education, Keimyung University, Daegu 42601, Republic of Korea; myongwonseo@kmu.ac.kr; 2 Department of Exercise Science, Falk College of Sport, Syracuse University, Syracuse, NY 13244, USA

**Keywords:** body shape, anthropometric measures, surrogate markers of insulin resistance, CVD, diabetes

## Abstract

**Highlights:**

**What are the main findings?**

- ABSI showed moderate discriminative ability for prevalent diabetes and cardiovascular disease in Korean adults.
- Higher ABSI categories were associated with greater odds of prevalent diabetes and cardiovascular disease.

**What is the implication of the main finding?**

- ABSI may serve as a simple anthropometric marker that complements conventional anthropometric measures in identifying adults with a greater burden of obesity-related cardiometabolic disease.

**Abstract:**

**Background/Objectives**: A body shape index (ABSI) has been proposed as an anthropometric measure reflecting abdominal body shape beyond body mass index (BMI). However, its relative cross-sectional discriminative ability compared with conventional anthropometric measures and surrogate markers of insulin resistance for prevalent diabetes and cardiovascular disease (CVD) remains poorly characterized in Korean adults. **Methods**: This cross-sectional study included 11,448 Korean adults aged 18–64 years from the 2019–2021 Korean National Health and Nutrition Examination Survey (KNHANES). Using receiver operating characteristic (ROC) curve analyses, we compared the discriminative ability of ABSI with BMI, waist circumference, waist-to-height ratio, homeostatic model assessment for insulin resistance (HOMA-IR), triglyceride-to-HDL cholesterol ratio (TG/HDL-C), and the triglyceride–glucose index (TyG) for prevalent diabetes and CVD. Multivariable logistic regression analyses assessed associations after adjustment for demographic and socioeconomic factors. **Results**: ABSI showed the highest ROC-AUC for both prevalent diabetes (74%) and CVD (74%) among the compared indices. In multivariable-adjusted analyses, individuals in the very high ABSI risk group had higher odds of prevalent CVD (OR, 3.75; 95% CI, 1.60–8.78) than those in the very low-risk group. Among individuals with diabetes, the odds of comorbid CVD were also higher in the very high ABSI group (OR, 3.38; 95% CI, 2.26–5.07). Similar patterns appeared in obesity-subgroup analyses. **Conclusions**: ABSI showed greater discriminative ability for prevalent diabetes and CVD than several conventional measures and insulin-resistance markers, supporting its potential utility as a simple marker of cardiometabolic disease burden. Prospective studies are needed to determine its prognostic value.

## 1. Introduction

The prevalence of obesity is reaching a global epidemic and has approximately tripled since 1975, contributing to a major public health problem worldwide [1]. Overweight and obesity are independent risk factors for diabetes and cardiovascular disease (CVD) [2]. For this reason, identifying an accurate estimator of adiposity is clinically important for identifying individuals with a higher burden of cardiometabolic disease. Traditionally, anthropometric measures including body mass index (BMI) and waist circumference (WC) are widely used as screening tools for estimating the degree of obesity [3,4,5]. However, these tools have limitations as they do not differentiate between fat mass, visceral adiposity, and fat-free mass [6,7]; so, they may not accurately reflect and provide a sufficient assessment of the cardiometabolic disease risks associated with increased adiposity [8]. Notably, we recently demonstrated that cardiometabolic diseases such as hypertension, dyslipidemia, and diabetes are not solely dependent on obesity status as defined by BMI [9]. Therefore, it is important to explore and develop simpler and more accessible tools for assessing obesity/adiposity that can be used in clinical practice. Compared with direct body composition assessments such as DXA, CT, and MRI, ABSI can be calculated easily from routine anthropometric measurements without specialized equipment or technical expertise, which may enhance its practicality in large-scale or screening-oriented settings [5,6,10]. Because ABSI incorporates waist circumference in relation to height and weight, it may provide additional information on body shape and central fat distribution beyond conventional measures such as BMI, WC, and WHtR.

A body shape index (ABSI), derived from height, weight, and WC, has recently been considered a potentially useful anthropometric indicator associated with obesity-related comorbidities [11,12]. In contrast to BMI, which primarily reflects overall body size, ABSI is a complementary anthropometric measure that incorporates BMI and height to better capture body shape and fat distribution. Krakauer & Krakauer reported in a study of U.S. adults that the hazard ratio (HR) per standard deviation (SD) in ABSI is 33% (95% CI; 1.20–1.48), while BMI (HR [95% CI]; 0.98 [0.89–1.08] and WC (1.07 [0.98–1.16]) did not show a significant association with increased risk of mortality [11]. In addition, a meta-analysis demonstrated that an increase in mortality risk associated with an SD increase in ABSI is 49% (95% CI [0.23, 0.81]; I^2^ = 98.5%) and 34% (95% CI [0.13, 0.58]; I^2^ = 98.8%) higher than that associated with BMI and WC, respectively [12]. These findings suggest that ABSI may provide prognostic information beyond BMI and WC in some populations.

ABSI is easy to calculate in clinical settings and may provide useful information on obesity-related morbidity and mortality [13,14,15]. However, it remains unclear which anthropometric adiposity measure shows the greatest discriminative ability for prevalent cardiometabolic disease in a large population-based sample of Korean adults. To date, few studies have directly compared the association and discriminative ability of ABSI with other anthropometric measures and surrogate indices of insulin resistance in a large population-based sample of Korean adults. Therefore, the purpose of this study was to examine the association and discriminative ability of ABSI for prevalent diabetes and CVD in Korean adults using nationally representative survey data. In addition, we compared ABSI with conventional anthropometric measures, including BMI, WC, and waist-to-height ratio (WHtR), as well as surrogate markers of insulin resistance, including HOMA-IR, TG/HDL-C, and TyG.

## 2. Materials and Methods

### 2.1. Data Source

This cross-sectional study utilized a large population-based dataset known as the Korean National Health and Nutrition Examination Survey (KNHANES) from 2019 to 2021. The KNHANES is conducted annually by the Korea Centers for Disease Control and Prevention (KCDC), which includes comprehensive and nationally representative data on health information, lifestyle, and nutritional factors. To ensure a representative sample, a two-stage stratified cluster sampling method was employed, using enumeration districts and households as the primary and secondary sampling units, respectively. Enumeration districts were stratified based on the first-level criteria (region, urban/rural classification, and housing type), second-level criteria (proportion of residential area), and implicit stratification criteria (proportion of household heads with different education levels). Within each selected district, 25 sample households were chosen through systematic sampling, excluding facilities such as nursing homes, military bases, prisons, and households with foreign nationals. The KNHANES was approved by the Institutional Review Board of the KDCA (Approval No. 2018-01-03-C-A, 2018-01-03-2C-A, and 2018-01-03-5C-A). This study was conducted in accordance with the ethical standards of the Declaration of Helsinki. Informed consent was obtained from all participants prior to participation in this study, and all participants signed an informed consent form before the survey and blood collection.

### 2.2. Study Population

We initially obtained KNHANES data from 2019 (*N* = 8110), 2020 (*N* = 7359), and 2021 (*N* = 7090). The present study focused on Korean adults aged 18–64 years. Adults aged 65 years or older were not included because the aim of this study was not to evaluate anthropometric indices in older adults, in whom age-related changes in stature, body composition, comorbidity burden, and medication use may complicate interpretation. Of the total pooled sample (*N* = 22,559), younger individuals (<18 years; *n* = 3721) and older adults (≥65 years; *n* = 5163) were excluded. Additionally, individuals with underweight and missing data on selected variables were excluded from the analysis (*n* = 2227). Finally, a total of 11,448 Korean adults were included in the final analysis.

### 2.3. A Body Shape Index (ABSI)

ABSI was calculated by using a simple formula: $ABSI=\frac{WC}{{BMI}^{2/3}\times {\;height}^{1/2}}$ [11]. Additionally, to control for the sex difference in ABSI, we converted to z-score using each ABSI mean and standard deviation (SD) of each sex for estimating the risk of cardiovascular disease (ABSIz-score = (ABSI − ABSImean (sex)/ABSISD (sex)). We applied the ABSI z-score quintile classification proposed by Krakauer et al. [11]: ABSIz-score < −0.868—very low risk; ABSIz-score −0.868 to −0.271—low-risk level; ABSIz-score −0.272 to 0.229—average risk; ABSIz-score 0.230 to 0.798—high risk; and >0.798—very high risk [11].

### 2.4. Demographic Information and Cardiometabolic Disease Risk

Demographic information included age, sex, household income level (quartile), education level (elementary, middle, high, undergraduate), current smoking status (never smoked, former smoker, smoker), family health history (hypertension, dyslipidemia, ischemic heart disease, stroke, diabetes, thyroid disease, hepatitis B), and occupation (administrators, managers, and professionals; office workers, service workers and shop sales workers; skilled agricultural and fishery workers; machine operators and assemblers, labors; jobless). Family history was considered positive if at least one of the following conditions was reported among family members: hypertension, dyslipidemia, ischemic heart disease, stroke, diabetes, thyroid disease, or hepatitis B. Physical characteristics included height, weight, body mass index (BMI) and WC. WHtR was calculated as waist (cm) divided by height (cm). BMI categories were defined using cut-off values of 23 kg/m^2^ for overweight and 25 kg/m^2^ for obesity [16]. Cardiometabolic characteristics included systolic blood pressure (SBP), diastolic blood pressure (DBP), mean arterial pressure (MAP; DBP + 0.4 × [SBP − DBP]) [17], HDL-C, triglycerides (TG), fasting plasma glucose (FPG), fasting insulin (FI), and HbA1c. Surrogate markers of insulin resistance were calculated using the homeostatic model assessment for insulin resistance (HOMA-IR; FPG [mg/dL] × FI [µU/mL]/405) [18], TG/HDL-C, and triglyceride–glucose index (TyG; ln (TG [mg/dL] × FPG [mg/dL]/2). CVD was defined using survey variables indicating self-reported physician-diagnosed hypertension, stroke, myocardial infarction, or angina pectoris. Accordingly, the CVD variable should be interpreted as a broad survey-based cardiovascular disease composite rather than an adjudicated clinical endpoint. Diabetes was defined by physician diagnosis, FPG ≥ 126 mg/dL, HbA1c ≥ 6.5%, use of antidiabetic medication, or insulin treatment.

### 2.5. Statistical Analysis

To compare the discriminative ability of anthropometric measures and surrogate markers of insulin resistance for prevalent diabetes and CVD, the area under the receiver operating characteristic curve (ROC-AUC) analysis was performed. Specifically, we clarified that the cut-off values were identified using the Youden Index to optimize sensitivity and specificity, and that post hoc analyses were conducted using the De-Long test. Multivariable logistic regression analyses were conducted to examine the associations of ABSI z-score quintile categories with prevalent diabetes and CVD by controlling for age, sex, household income levels, education levels, current smoking status, family health history, and occupation. Before conducting ANCOVA, the assumptions of normality and homogeneity of variance were assessed to confirm the appropriateness of the analysis. The differences in age, height, weight, BMI, cardiometabolic disease risk factors, and surrogate markers of insulin resistance between obesity status were analyzed by ANCOVA (adjusted for age and sex), followed by Bonferroni correction for multiple comparisons. In addition, chi-square tests were used to compare categorical variables (i.e., disease status). The continuous data were expressed as the mean, and standard error of the mean, while categorical data were presented as frequency and percentage. The significant level was set at 0.05. Data were analyzed with SPSS (version 28, SPSS Inc., Chicago, IL, USA).

## 3. Results

### 3.1. Anthropometric, Cardiometabolic Disease Risk Factors, Surrogate Markers of Insulin Resistance, and Disease Status

Table 1 presents the anthropometric, metabolic, and disease characteristics of participants according to weight status (normal weight, overweight, and obesity). All anthropometric measures, including BMI, WC, WHtR, and ABSI, increased progressively from the normal-weight group to the overweight group and to the obesity group. All blood-pressure variables, including SBP, DBP, and MAP, gradually increased from normal-weight to overweight and to obese. HDL-C progressively decreased from obese to overweight and to normal-weight. In addition, TG, FPG, FI, HbA1c, HOMA-IR, TG/HDL-C, and TyG index progressively worsened from the normal-weight group to the overweight group and to the obesity group.

### 3.2. Associations of ABSI to CVD and Diabetes

Multivariable logistic regression analyses were performed to assess the odds ratios and 95% CI for prevalent CVD and diabetes across ABSI categories (Table 2). Compared with individuals in the very-low-ABSI-risk group, the odds of prevalent CVD were higher in the average-risk (OR, 2.99; 95% CI, 1.26–7.11), high-risk (OR, 3.27; 95% CI, 1.39–7.13), and very-high-risk groups (OR, 3.75; 95% CI, 1.60–8.78). Similarly, among individuals with diabetes, the odds of comorbid prevalent CVD were higher in the average-risk (OR, 1.80; 95% CI, 1.18–2.74), high-risk (OR, 2.50; 95% CI, 1.65–3.78), and very-high-risk groups (OR, 3.38; 95% CI, 2.26–5.07) than in the very-low-risk group.

### 3.3. The Discriminative Performance of the ABSI for Diabetes and CVD

The discriminative performance of ABSI for prevalent diabetes and CVD is presented in Table 3. In the total cohort, the ROC-AUC of ABSI for prevalent diabetes was 0.740 (sensitivity, 70.6%; specificity, 65.8%), and the ROC-AUC for prevalent CVD was 0.740 (sensitivity, 77.0%; specificity, 59.1%). ABSI showed the highest ROC-AUC among the compared anthropometric measures and surrogate markers of insulin resistance, including WHtR, WC, BMI, TyG, TG/HDL-C, and HOMA-IR, for both prevalent diabetes and CVD. In subgroup analyses restricted to individuals with obesity, ABSI also showed the highest ROC-AUC for prevalent diabetes (0.690) and CVD (0.690) among the compared indices (Table 4). Overall, similar patterns were observed in both the total cohort and subgroup analyses.

## 4. Discussion

In this nationally representative cross-sectional sample of Korean adults, ABSI showed greater discriminative ability for prevalent diabetes and CVD than several conventional anthropometric measures and surrogate markers of insulin resistance. Higher ABSI categories were also associated with a greater burden of prevalent diabetes and CVD after multivariable adjustment. These findings suggest that ABSI may be a practical anthropometric marker of prevalent cardiometabolic disease in Korean adults. Importantly, however, the present results should be interpreted as cross-sectional evidence of association and discrimination rather than evidence of causality or longitudinal prediction. Accordingly, ABSI should not be interpreted as a prognostic marker on the basis of the present findings alone.

The present study adds to the literature by showing that ABSI had the highest discriminative ability for prevalent diabetes and CVD among the compared indices in this nationally representative sample of Korean adults. A similar pattern was also observed; ABSI, compared with other anthropometrics, showed the highest discriminative performance for diabetes and CVD even when we limited the data to individuals with obesity. In clinical practice, simple and accessible anthropometric markers may help identify individuals with a greater burden of prevalent cardiometabolic disease. Although ABSI showed the highest ROC-AUC values among the compared indices, the absolute differences were modest. Therefore, the clinical significance of these differences should be interpreted cautiously. In practice, these findings do not suggest that ABSI should replace established diagnostic or clinical assessments for diabetes or CVD. Rather, ABSI may be considered a simple complementary anthropometric marker that provides additional cross-sectional information on cardiometabolic disease burden. Although BMI is widely used as a general anthropometric indicator of obesity, abdominal adiposity may be more closely associated with diabetes and CVD than total adiposity alone [19], as demonstrated by growing evidence [20,21]. However, although WC reflects abdominal adiposity, it also overlaps with BMI as a measure of overall body size. In this context, ABSI has been introduced as a comprehensive anthropometric index that accounts for height, weight, and WC, capturing body shape and fat distribution more specifically.

A previous study showed that ABSI (ROC-AUC [95% CI], male: 73% [0.71–0.75], female: 73% [0.69–0.77]) had a higher discriminative performance for CVD, including coronary heart disease and cerebrovascular disease, compared to BMI (male: 57% [0.55–0.59], female: 58% [0.53–0.62]) in Japanese male and female adults with a mean age of 52 years [22]. Additionally, Zhao et al. demonstrated that ABSI (male: 64.9% [0.62–0.68], female: 69.8% [0.68–0.72]) had a better discriminative performance for diabetes compared to BMI (male: 61.9% [0.59–0.64], female: 64.5% [0.62–0.67]) in Chinese male and female adults aged 48 (39 to 58 years) [23]. We speculate that incorporating overall adiposity status into regression allometry for fat distribution improves the discriminative performance for cardiometabolic disease. Therefore, although ABSI cannot be interpreted as a prognostic factor due to the cross-sectional design, it may represent a practical anthropometric marker associated with prevalent diabetes and CVD.

To our knowledge, few studies have evaluated the association and discriminative ability of ABSI for prevalent cardiometabolic disease in a large population-based sample of Korean adults. To address this gap, we conducted multivariable logistic regression analyses to examine the association between ABSI and prevalent diabetes and CVD. Bozorgmanesh et al. demonstrated that ABSI was higher in multivariate-adjusted HR for incident CVD, including coronary heart disease, stroke, and cerebrovascular mortality, in both Iranian males and females (male; 1.26 [1.09–1.46], female; 1.17 [1.03–1.32]) than BMI (male; 1.06 [0.94–1.20], female; 1.02 [0.90–1.16]), WC (male; 1.15 [1.03–1.28], female; 1.11 [0.98–1.27]), and WHtR (male; 1.16 [1.02–1.31], female; 1.14 [0.99–1.03]) [24]. Additionally, a study with 5.4 years of follow-up among non-obese Chinese adults aged ≥35 years showed that that ABSI is associated with a 30% greater risk of CVD mortality (HR: 1.30 per 0.01-unit increase; 95% CI: 1.08–1.58) [25]. Our findings therefore extend the literature by supporting the potential value of considering both total adiposity and body fat distribution when identifying prevalent cardiometabolic disease burden.

An additional finding of the present study was that the prevalence of cardiometabolic disease and the severity of insulin resistance increased across higher ABSI categories (by using quintile classification). A previous study demonstrated that the prevalence of diabetes gradually increased with increasing ABSIz-score risk levels in both males and females: (males—1st quartile: 6.5%, 2nd quartile: 7.6%, 3rd quartile: 11%, 4th quartile: 14.7%; females—1st quartile: 7.9%, 2nd quartile: 10.1%, 3rd quartile: 12.4%, 4th quartile: 12.9%) among Chinese adults aged ≥ 35 years [26]. Grant et al., who investigated 3311 Australian adults aged over 18 years, found that out of the total 374 obesity-related mortalities, the highest ABSI quartile (*n* = 244) and 3rd ABSI quartile (*n* = 79) accounted for 86.3% of the total deaths [27]. In addition, in a longitudinal large population-based study among healthy Korean adults middle-aged (40.9 years), the prevalence of diabetes and HOMA-IR increased across higher ABSI categories from the ABSI z-score quintile increased [28]. The prevalence of diabetes and cardiovascular disease was markedly elevated in the fifth ABSI quintile relative to the first quintile, indicating a positive association between higher ABSI categories and prevalent disease burden. An additional finding of the present study was that higher ABSI categories were associated with a greater burden of prevalent cardiometabolic disease. This graded pattern was consistent with prior studies reporting positive associations of higher ABSI levels with diabetes, mortality, and cardiometabolic risk. Together, these findings suggest that ABSI may provide complementary anthropometric information on prevalent disease burden. These findings were consistent with the results observed in the total cohort population. Therefore, these findings suggest that ABSI may be a clinically useful anthropometric marker associated with prevalent diabetes and CVD in both the total cohort and subgroup analyses.

The strengths of the present investigation include: (1) a comprehensive assessment of the association and discriminative ability of ABSI for prevalent cardiometabolic disease in a large population-based sample of Korean adults; (2) a simultaneous comparison of anthropometric measures and surrogate markers of insulin resistance; and (3) additional subgroup analyses among individuals with obesity. Nevertheless, several limitations should be acknowledged. First, because of the cross-sectional design, the observed associations between ABSI and prevalent cardiometabolic disease do not permit causal inference and should not be interpreted as evidence of longitudinal or prognostic utility. Second, although ABSI showed the highest ROC-AUC values among the compared indices, the absolute differences were modest; therefore, their clinical significance should be interpreted cautiously. These findings do not suggest that ABSI should replace established diagnostic or clinical assessments for diabetes or CVD, but rather that it may provide complementary anthropometric information on cardiometabolic disease burden. Third, residual confounding is possible because, although the models were adjusted for several demographic and socioeconomic variables, other relevant lifestyle and clinical factors, including physical activity, dietary intake, alcohol consumption, medication use beyond diabetes treatment, and additional cardiovascular risk factors, may also have influenced both anthropometric measures and prevalent cardiometabolic disease. Fourth, although ABSI was standardized using sex-specific z-scores, the primary analyses did not fully address potential sex-specific differences in the associations of ABSI with diabetes and CVD. Finally, because the present study was designed to evaluate Korean adults aged 18–64 years, the findings should not be generalized to other age groups, including older adults aged 65 years or older, or to other racial or ethnic populations without caution. Therefore, further studies are needed to validate these findings in older adults and diverse populations and to determine whether similar associations are observed over time.

## 5. Conclusions

ABSI showed greater discriminative ability for prevalent diabetes and CVD than several conventional anthropometric measures and surrogate markers of insulin resistance in Korean adults aged 18–64 years. Higher ABSI categories were also associated with a greater burden of prevalent cardiometabolic disease. These findings support the potential role of ABSI as a complementary anthropometric marker associated with cardiometabolic disease burden in Korean adults. However, prospective studies are needed to determine whether ABSI has prognostic value for incident disease.

## Figures and Tables

**Table 1:** Anthropometric characteristics, cardiometabolic risk factors, and disease status of participants according to weight status (mean ± SEM).

Variables	Normal-Weight [A] (*n* = 4558)	Overweight [B] (*n* = 2603)	Obese [C] (*n* = 4287)	ANCOVA *p*-Value	Post Hoc	$\eta_{p}^{2}$
Anthropometric						
BMI (kg/m^2^)	21.1 ± 0.02	24.0 ± 0.01	28.1 ± 0.04	<0.01	A < B < C	0.70
Waist circumference (cm)	75.1 ± 0.1	83.5 ± 0.1	93.5 ± 0.1	<0.01	A < B < C	0.59
WHtR	0.46 ± 0.001	0.50 ± 0.001	0.56 ± 0.001	<0.01	A < B < C	0.60
ABSI	0.077 ± 0.0001	0.0780 ± 0.0001	0.079 ± 0.0001	<0.01	A < B < C	0.01
Cardiometabolic characteristics						
SBP (mmHg)	111.7 ± 0.2	116.7 ± 0.3	121.8 ± 0.2	<0.01	A < B < C	0.06
DBP (mmHg)	73.0 ± 0.1	75.9 ± 0.2	79.6 ± 0.2	<0.01	A < B < C	0.06
MAP (mmHg)	88.5 ± 0.2	92.2 ± 0.2	96.5 ± 0.2	<0.01	A < B < C	0.07
HDL-C (mg/dL)	57.8 ± 0.2	51.9 ± 0.2	48.2 ± 0.2	<0.01	C < B < A	0.07
TG (mg/dL)	101.5 ± 1.2	136.2 ± 2.4	165.2 ± 2.0	<0.01	A < B < C	0.04
FPG (mg/dL)	94.8 ± 0.2	99.4 ± 0.4	106.2 ±0.4	<0.01	A < B < C	0.04
FI (µU/mL)	6.6 ± 0.1	8.6 ± 0.1	13.1 ± 0.2	<0.01	A < B < C	0.12
HbA1c	5.57 ± 0.01	5.75 ± 0.02	5.99 ± 0.02	<0.01	A < B < C	0.03
Insulin resistance indices						
HOMA-IR	1.57 ± 0.02	2.2 ± 0.0	3.5 ± 0.1	<0.01	A < B < C	0.10
TG/HDL-C	1.98 ± 0.03	2.99 ± 0.07	3.86 ± 0.06	<0.01	A < B < C	0.04
TyG index	8.3 ± 0.0	8.6 ± 0.0	8.9 ± 0.0	<0.01	A < B < C	0.10
Disease status				Chi-square		
CVD (*n*, %)	358 (8)	359 (14)	1043 (24)	<0.01		
Diabetes (*n*, %)	172 (4)	156 (6)	417 (10)	<0.01		

Note: BMI: body mass index, WHtR: waist-to-height ratio, ABSI: a body shape index, SBP: systolic blood pressure, DBP: diastolic blood pressure, MAP: mean arterial pressure, HDL-C: high-density lipoprotein cholesterol, TG: triglyceride, FPG: fasting plasma glucose, Fi: fasting insulin, HbA1c: hemoglobin A1c, HOMA-IR: homeostatic model assessment for insulin resistance, TyG index: triglyceride–glucose index, ANCOVA: adjusted for age and sex.

**Table 2:** Associations of prevalent cardiometabolic diseases with ABSI based on logistic regression analyses in the total cohort.

ABSI Z-Score		Number of Disease Cases	95% CI	Odds Ratio	*p*-Value
CVD	Very low risk	6		1.000	
	Low risk	21	0.793–4.965	1.984	0.14
	Average risk	44	1.255–7.108	2.986	<0.05
	High risk	57	1.385–7.127	3.271	<0.01
	Very high risk	107	1.597–8.783	3.746	<0.01
Diabetes	Very low risk	29		1.000	
	Low risk	60	0.795–1.976	1.254	0.33
	Average risk	116	1.177–2.741	1.796	<0.01
	High risk	180	1.650–3.777	2.504	<0.01
	Very high risk	360	2.255–5.071	3.381	<0.01

Note: CVD: cardiovascular disease, Adjusted for age, sex, education, smoking, household income level and occupation, family health history. Odds ratios and 95% confidence intervals were estimated using multivariable logistic regression analysis. Models were adjusted for age, sex, education, smoking, household income level, occupation, and family health history. CVD, cardiovascular disease.

**Table 3:** Area under the receiver operating characteristic (ROC) curve of the various anthropometric measures and surrogate markers of insulin resistance for CVD and diabetes in the total cohort.

	Indicator	ROC Curve	95% CI	Sensitivity (%)	Specificity (%)	Cut-Off	Post Hoc (*p*-Value)					
							HOMA-IR	TG/HDL-C	TyG	BMI	WC	WHtR
CVD	HOMA-IR	0.639 ***	0.603–0.675	60.0	61.0	2.13						
	TG/HDL-C	0.611 ***	0.578–0.644	63.4	54.7	2.20	0.145					
	TyG	0.631 ***	0.598–0.665	55.3	65.2	8.80	0.675	<0.05				
	BMI	0.626 ***	0.594–0.659	71.1	50.4	23.89	0.501	0.446	0.81			
	WC	0.695 ***	0.666–0.723	83.4	47.6	82.5	<0.01	<0.01	<0.01	<0.01		
	WHtR	0.704 ***	0.675–0.732	89.4	42.6	0.49	<0.01	<0.01	<0.01	<0.01	0.33	
	ABSI	0.740 ***	0.711–0.768	77.0	59.1	0.79	<0.01	<0.01	<0.01	<0.01	<0.01	<0.05
Diabetes	HOMA-IR	0.693 ***	0.673–0.714	60.3	70.3	2.45						
	TG/HDL-C	0.638 ***	0.620–0.657	79.2	42.8	1.71	<0.01					
	TyG	0.733 ***	0.715–0.750	72.1	63.0	8.73	<0.01	<0.01				
	BMI	0.627 ***	0.607–0.647	69.1	51.3	23.88	<0.01	0.35	<0.01			
	WC	0.691 ***	0.673–0.709	69.5	59.8	85.5	0.82	<0.01	<0.01	<0.01		
	WHtR	0.714 ***	0.697–0.731	84.6	47.3	0.50	<0.05	<0.01	0.08	<0.01	<0.01	
	ABSI	0.740 ***	0.723–0.758	70.6	65.8	0.79	<0.01	<0.01	0.53	<0.01	<0.01	<0.01

Note: HOMA-IR: homeostatic model assessment of insulin resistance, TG: triglyceride, HDL-C: high-density lipoprotein cholesterol, TyG index: triglyceride–glucose index, BMI: body mass index, WC: waist circumference, WHtR: waist-to-height ratio, ABSI: a body shape index. ROC-AUC values were obtained from receiver operating characteristic curve analyses, and post hoc comparisons between ROC curves were performed using the DeLong test. *** *p* < 0.001.

**Table 4:** Area under the receiver operating characteristic (ROC) curve of various anthropometric measures and surrogate markers of insulin resistance for prevalent CVD and diabetes among individuals with obesity (*N* = 4287).

	Indicator	ROC Curve	95% CI	Sensitivity (%)	Specificity (%)	Cut-Off	Post Hoc (*p*-Value)					
							HOMA-IR	TG/HDL-C	TyG	BMI	WC	WHtR
CVD	HOMA-IR	0.578 **	0.527–0.629	47.8	69.2	3.62						
	TG/HDL-C	0.529	0.482–0.576	97.0	9.9	1.18	0.10					
	TyG	0.555 *	0.508–0.603	56.0	55.8	8.93	0.41	<0.05				
	BMI	0.480	0.431–0.530	93.3	9.1	25.3	<0.01	0.15	<0.05			
	WC	0.586 ***	0.539–0.633	35.8	77.3	98.5	0.80	0.06	0.29	<0.01		
	WHtR	0.605 ***	0.560–0.650	57.5	59.8	0.57	0.36	<0.05	0.10	<0.01	0.31	
	ABSI	0.690 ***	0.647–0.733	74.6	54.8	0.79	<0.01	<0.01	<0.01	<0.01	<0.01	<0.01
Diabetes	HOMA-IR	0.660 ***	0.632–0.688	66.2	59.3	3.00						
	TG/HDL-C	0.550 ***	0.522–0.577	71.7	38.0	2.28	<0.01					
	TyG	0.672 ***	0.645–0.698	76.0	50.0	8.81	0.46	<0.01				
	BMI	0.523	0.494–0.552	28.1	76.6	29.39	<0.01	0.18	<0.01			
	WC	0.611 ***	0.584–0.638	82.5	34.0	89.5	<0.01	<0.01	<0.01	<0.01		
	WHtR	0.649 ***	0.623–0.675	74.8	47.9	0.55	0.51	<0.01	0.23	<0.01	<0.01	
	ABSI	0.690 ***	0.664–0.717	68.1	60.8	0.79	0.10	<0.01	0.32	<0.01	<0.01	<0.01

Note: HOMA-IR: homeostatic model assessment of insulin resistance, TG: triglyceride, HDL-C: high-density lipoprotein cholesterol, TyG index: triglyceride–glucose index, SPISE: single point insulin sensitivity estimator, BMI: body mass index, WC: waist circumference, WHtR: waist-to-height ratio, ABSI: a body shape index. ROC-AUC values were obtained from receiver operating characteristic curve analyses, and post hoc comparisons between ROC curves were performed using the DeLong test. * *p* < 0.05, ** *p* < 0.001, *** *p* < 0.001.

## Data Availability

The data that support the findings of this study are publicly available from the Korea National Health and Nutrition Examination Survey (KNHANES) and can be accessed through the official KNHANES website https://knhanes.kdca.go.kr; (accessed on 15 May 2023).

## Abbreviations

ABSI	a body shape index
BMI	body mass index
CVD	cardiovascular disease
KNHANES	Korean National Health and Nutrition Examination Survey
ROC-AUC	area under the receiver operating characteristic curve
WC	waist circumference
HOMA-IR	homeostatic model assessment for insulin resistance
TG	triglyceride
HDL-C	high-density lipoprotein cholesterol
TyG	triglyceride–glucose index
HR	hazard ratio
SD	standard deviation
KCDC	Korea Centers for Disease Control and Prevention
SBP	systolic blood pressure
DBP	diastolic blood pressure
MAP	mean arterial pressure
WHtR	waist-to-height ratio
FPG	fasting plasma glucose
FI	fasting insulin
HbA1c	hemoglobin A1c
